# A novel platform for engineered AAV-based vaccines

**DOI:** 10.1016/j.omtm.2025.101418

**Published:** 2025-01-22

**Authors:** Sabrina Babutzka, Miranda Gehrke, Anastasia Papadopoulou, Maria Diedrichs-Möhring, Maria Giannaki, Lena Hennis, Bastian Föhr, Cale Kooyman, Andreas Osterman, Evangelia Yannaki, Gerhild Wildner, Hermann Ammer, Stylianos Michalakis

**Affiliations:** 1 Department of Ophthalmology, University Hospital, LMU Munich, 80336 Munich, Germany; 2 Hematology Department-Hematopoietic Cell Transplantation Unit, Gene and Cell Therapy Center, “George Papanikolaou” Hospital, 570 10 Thessaloniki, Greece; 3 Max Von Pettenkofer Institute and Gene Center, Virology, LMU Munich, 80336 Munich, Germany; 4 Department of Medicine, University of Washington, Seattle, WA 91895, USA; 5 Department of Veterinary Sciences, Ludwig-Maximilians-Universität München, 80539 Munich, Germany

**Keywords:** AAV, capsid, engineered capsid, vaccine, virus-like particles, protein carrier, humoral response, cellular response

## Abstract

Engineering of adeno-associated virus (AAV) capsids allowed for the development of gene therapy vectors with improved tropism and enhanced transduction efficiency. Capsid engineering can also be used to adapt the AAV technology for applications outside gene therapy. Here, we investigated modified AAV capsids as scaffolds for the presentation of large immunogenic antigens to elicit a strong and specific immune response against pathogens. Using SARS-CoV-2 as a model pathogen, we introduced ∼200 amino acids of the SARS-CoV-2 receptor-binding domain (RBD) into a surface-exposed variable loop region of AAV2 and AAV9, resulting in AAV2.RBD and AAV9.RBD capsids (AAV.RBDs). This engineering endowed AAV.RBDs with SARS-CoV-2-like properties, such as angiotensin-converting enzyme 2 receptor affinity. In line with this, AAV.RBDs were neutralized by sera from human donors vaccinated against SARS-CoV-2. When administered subcutaneously to rabbits, AAV.RBDs elicited a strong humoral response against SARS-CoV-2 RBD. Moreover, the AAV.RBDs were able to trigger RBD-specific cellular immune responses in peripheral human lymphocytes. In conclusion, this novel AAV-based next-generation vaccine platform allows for the presentation of large antigenic sequences to elicit strong and specific immune responses. This versatile vaccine technology could be explored in the context of diseases where conventional immunization approaches have been unsuccessful.

## Graphical abstract

**Figure:**
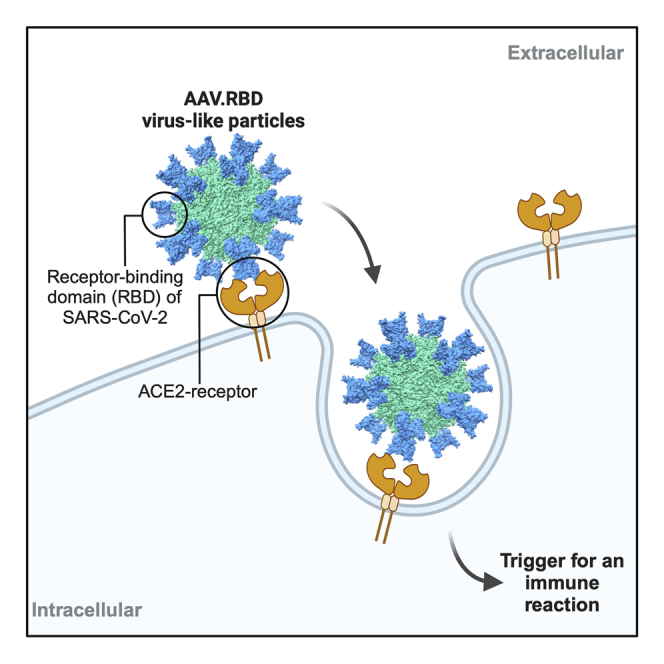

## Teaser

Michalakis and colleagues present a new vaccine platform technology based on modified adeno-associated virus (AAV) capsids that display large immunogenic sequences at the capsid surface. Genome-free virus-like particles (VLPs) based on modified AAV capsids that display the receptor-binding domain of SARS-CoV-2 elicited strong and specific immune responses.

## Introduction

Adeno-associated viruses (AAVs) are small, non-enveloped viruses that consist of an icosahedral capsid carrying a single-stranded DNA genome of approximately 4.7 kb. The AAV genome contains two genes: *Rep* and *Cap. Rep* encodes four non-structural Rep proteins, which are essential for viral genome transcription, replication, and shuttling into assembled AAV capsids.^1^ *Cap* encodes three capsid-forming structural viral proteins VP1, VP2, and VP3, as well as the non-structural proteins, AAP and MAAP, which support the assembly of the capsid^2^ or viral egress.^3^ On average, 5 VP1 assemble with 5 VP2 and 50 VP3 subunits to form the characteristic icosahedral AAV capsid with a diameter of approximately 25 nm.^1^ The structural VPs share the same C-terminal region within the Cap ORF but have an N-terminal region of varying length. AAVs are widely used as vectors for gene delivery, both in biomedical research and in clinical gene therapy products for the treatment of inherited or acquired diseases.^4^

Besides its use in various preclinical and clinical gene therapy approaches and currently eight authorized gene therapy products, the AAV technology has also been explored for vaccination strategies.^5^^,^^6^ Vaccines are unequivocally the most effective medical tool to protect from infectious diseases or to prevent severe complications by reducing morbidity and mortality.^7^ In the past decade, various next-generation technologies have been explored to develop novel vaccines that rely on nucleic acids, viral vectors, or virus-like particles (VLPs). The COVID-19 pandemic exposed the need to develop effective vaccines quickly and to manufacture them on a large scale. This led to various innovations in vaccinology, resulting in faster development strategies, production, and upscaling.^8^ The most commonly explored vaccination platforms included live attenuated^9^ or inactivated viruses,^10^ vectorized adenoviruses,^11^ and mRNA-based vaccines.^12^ Among those, the mRNA-based vaccine technology has proven particularly useful due to its flexibility to adapt to evolving SARS-CoV-2 genomic variants in a time- and cost-sensitive manner.^13^ However, mRNA-based vaccines require lipid nanoparticle formulations that are often not well tolerated and require strict storage at very low temperatures, presenting an obstacle for transport and distribution.^14^

Here, we explored modified AAV capsids as a scaffold for the presentation of large immunogenic epitopes to elicit a strong and specific immune response against pathogens. We introduced ∼200 amino acids (aa) of the SARS-CoV-2 receptor-binding domain (RBD) into the surface-exposed variable loop region VIII of AAV2 and AAV9, resulting in AAV2.RBD and AAV9.RBD capsids. We describe the construction of the capsid variants and the subsequent testing of their functionality and immunogenicity.

## Results

### Concept of VLPs introducing immunogens

**Figure 1:**
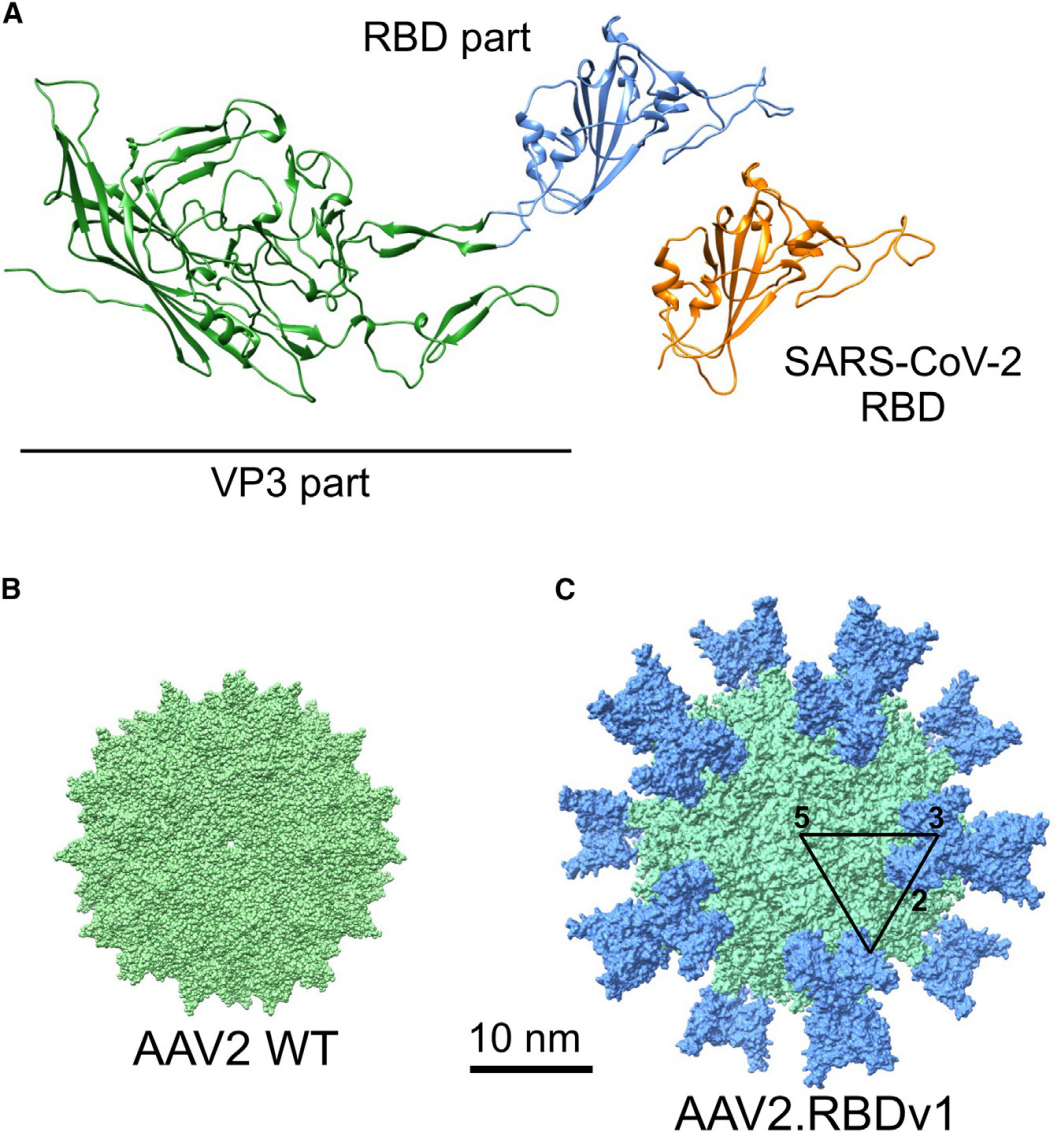
Predicted structure of engineered AAV.RBD capsid (A) AlphaFold2 *in-silico*-predicted single VP3 monomer of AAV2.RBDv1. The capsid-attached RBD domain (blue) adopts a similar structure as the SARS-CoV-2 RBD (orange, PDB: 6M0J). (B) Structure of the AAV2 60-mer capsid (light green, PDB: 8FZ0). (C) Predicted assembled 60-mer capsid structure based on the VP3 monomer shown in (A) shown from the 5-fold axis. The RBD domain is shown in blue. The AAV2 WT part is light green. The black triangle marks one icosahedral plane to illustrate the 5-fold (5), 3-fold (3), and 2-fold (2) symmetry axis.

Studies have shown that the SARS-CoV-2 RBD facilitates cell entry through binding to the human angiotensin-converting enzyme 2 receptor (ACE2) (Figure S1A).^15^^,^^16^ This RBD has been shown to be highly immunogenic^17^ and the vast majority of SARS-CoV-2-neutralizing antibodies (NAbs) are directed against the RBD.^18^^,^^19^^,^^20^^,^^21^ Therefore, we used the SARS-CoV-2 RBD as a model sequence to test the ability of AAV capsids to function as a scaffold for presenting large immunogenic sequences to elicit strong immune responses. To this end, we modified the AAV2 capsid by inserting the 197 aa SARS-CoV2 RBD into the surface-exposed variable loop region VIII of all three viral proteins (VP1-VP3) (Figure S1B). The insertion was made between positions N587 and R588 (VP1 numbering) in the common VP3 region, which is conserved in all three viral proteins (Figure S1C), resulting in a predicted structure as depicted in Figure 1A. The predicted quaternary structure of this modified capsid, which is referred to hereinafter as AAV2.RBDv1, retains the characteristic icosahedral shape with a conserved 2-, 3-, and 5-fold axis of symmetry and shows the RBD insertion structurally exposed 60 times at the capsid surface (Figures 1B and 1C). In addition, we generated another variant in which the inserted SARS-CoV2 RBD sequence was slightly shifted and longer (206 aa) to contain additional RBD-adjacent residues known to harbor immunogenic T cell epitopes, resulting in AAV2.RBDv2.^22^ To evaluate the impact of the AAV serotype, we generated a version of AAV2.RBDv1 in the context of AAV9 by inserting the 197 aa SARS-CoV2 RBD into the corresponding insertion site in AAV9 VP1 between positions N588 and A589.

### AAV.RBD vectors and genome-free VLPs

**Figure 2:**
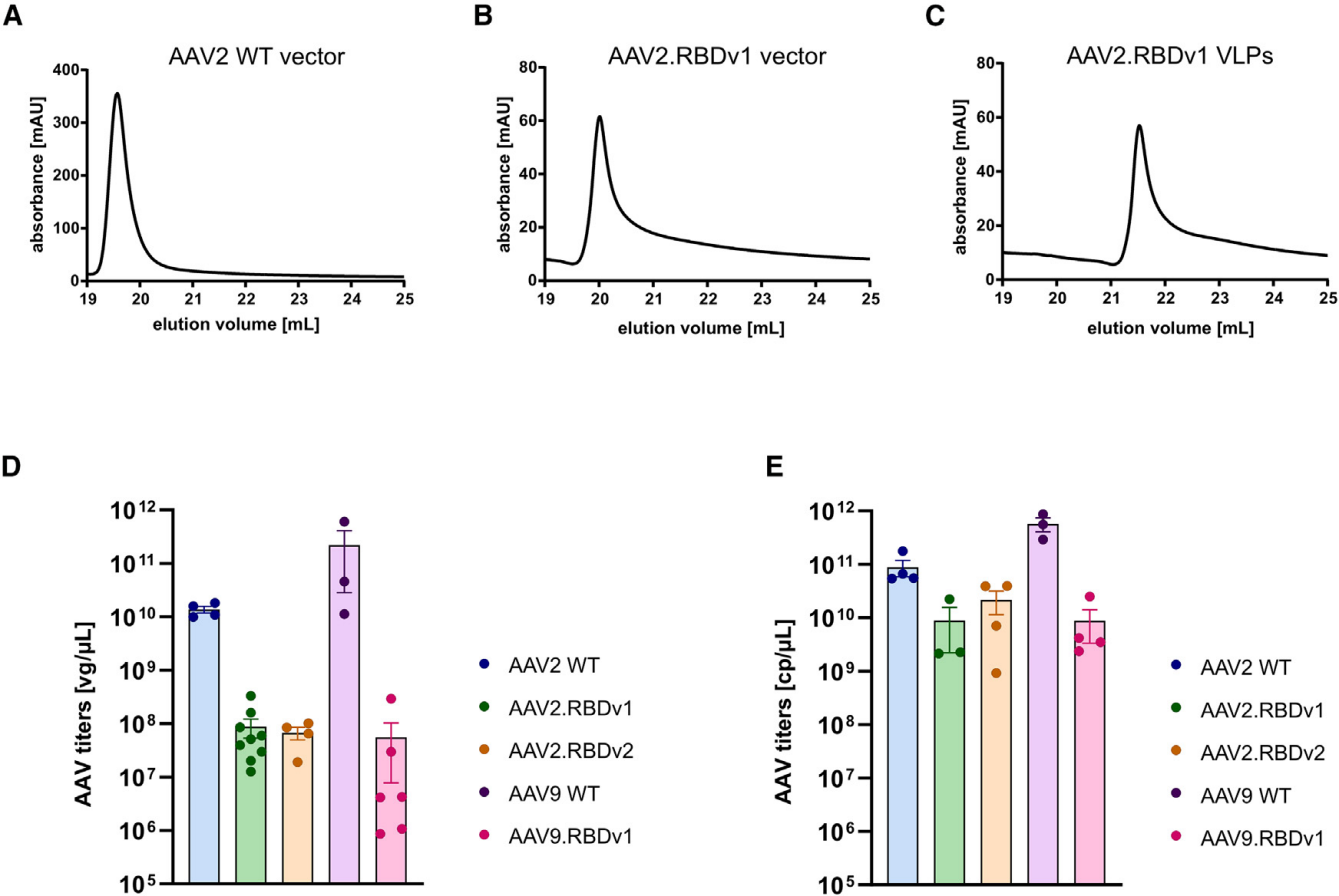
AAVx affinity chromatography and titers of AAV.RBDs (A–C) AAVx chromatograms showing the elution behavior of AAV2 WT vector (A), AAV2.RBDv1 vector (B), and AAV2.RBDv1 genome-free virus-like particles (VLPs) (C). The vector versions carried an ITR-flanked scCMV-eGFP reporter cassette. AAV2.RBDv1 VLPs were produced in the absence of a pTransgene ITR plasmid. (D) Genomic titer in vector genomes (vg)/μL of vector variants determined via ITR-directed real-time qPCR. (E) Capsid titer in capsid particles (cp)/μL measured by SLS. Bars indicate mean ± SEM.

To test whether the novel AAV.RBD capsids can be used to produce infectious particles, we produced them as AAV vectors containing a self-complementary genome expressing eGFP under the control of a CMV promoter (scCMV-eGFP) and evaluated their transduction properties in HEK293T cells. In addition, we produced genome-free VLPs that lack a vector genome. Interestingly, AAV.RBD could be affinity purified using AAVx affinity chromatography, confirming the AAV-like nature of genome-containing vector and empty VLP versions. The chromatograms obtained for genome-containing AAV2.RBDv1 vectors showed a similar shape, binding, and elution profile as conventional AAV2 wild-type (WT) vectors with only a slight shift toward later elution volume (Figures 2A and 2B). Empty AAV2.RBDv1 VLPs could also be purified with AAVx, but the chromatogram was further shifted toward higher elution volumes (Figure 2C). This suggests that AAV.RBD vector capsids carrying large peptide insertions still have specific AAV conformational epitopes to an extent to retain AAV capsid-like properties during affinity chromatography. However, the absorbance peak height and AUC were reduced, suggesting much lower genomic titers of the AAV2.RBDv1 vector variant compared with AAV2 WT. To follow this up, we performed static light scattering (SLS) measurements of capsid titers and, in the case of genome-containing vector variants, real-time quantitative PCR (qPCR) to determine genomic titers. Our production process yields genomic titers for AAV2 and AAV9 vectors in the range of 1–2 × 10^10^ and 1–2 × 10^11^ vg/μL, respectively. However, the mean genomic titers of the vectors produced with AAV2.RBDv1, AAV2.RBDv2, and AAV9.RBDv1 were two to three log units lower and ranged from 5 to 9 × 10^7^ vg/μL (Figure 2D). Capsid titers of the AAV.RBD variants were only one log unit lower than the corresponding WT vectors (Figure 2E). Thus, the full/empty ratio was much lower in the engineered vector variants than in the WT, suggesting a lower packaging capacity or reduced stability of AAV.RBDs. To test the stability of AAV.RBD variants, we performed intrinsic differential scanning fluorimetry of AAV2.RBDv2. We observed a mean melting temperature of 63°C for AAV2.RBDv2 compared with 68.3°C for AAV2 WT (Figure S4). This shows that AAV2.RBDv2 is slightly less thermostable than AAV2 WT, but still within the expected range for non-enveloped viruses.^23^

### AAV.RBD vectors show enhanced transduction levels in ACE2-OE-HEK293T cells

**Figure 3:**
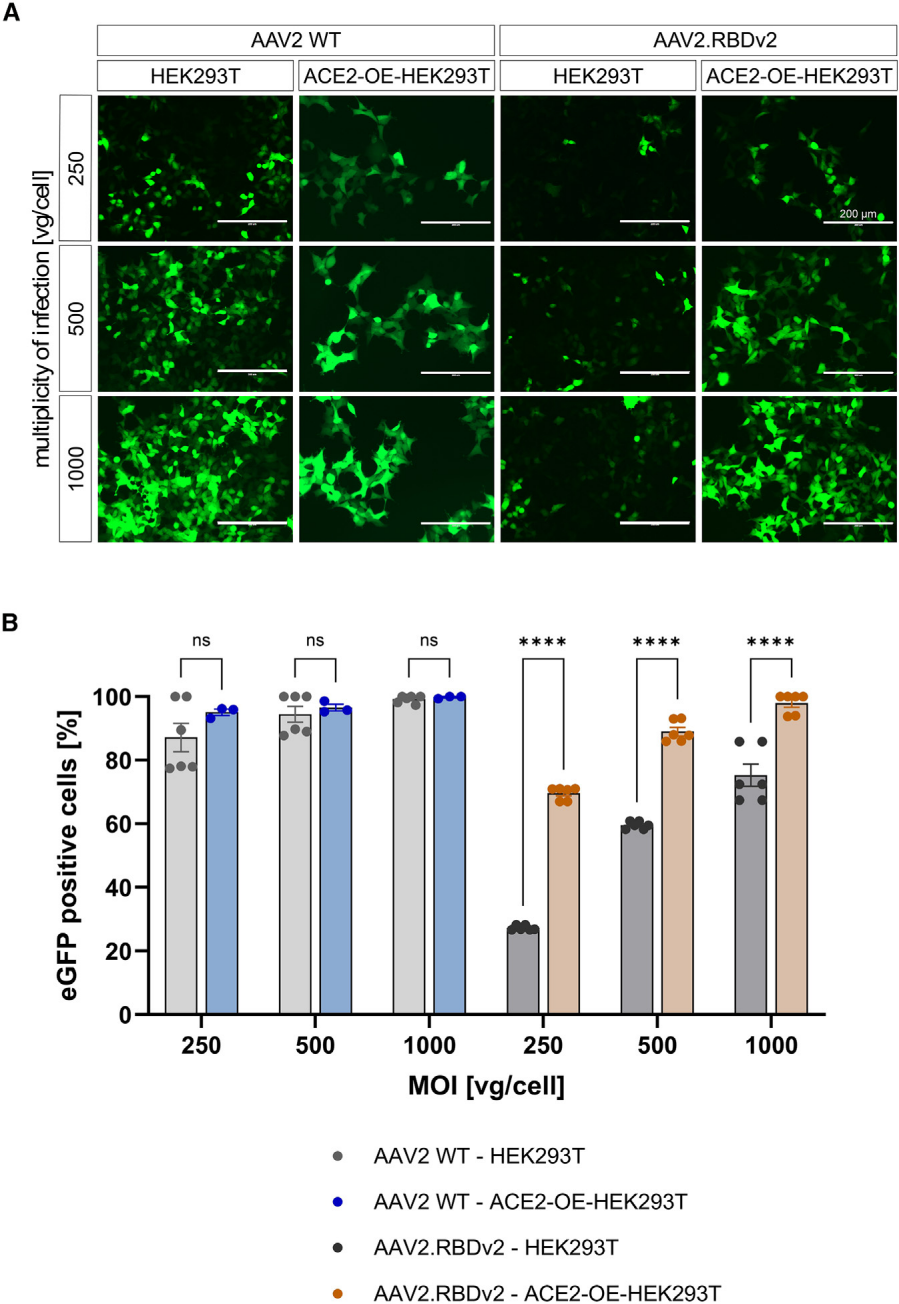
Transduction properties of AAV2 WT and AAV2.RBDv2 (A) Representative epifluorescence images of native HEK293T cells or HEK293T cells stably overexpressing ACE2 (ACE2-OE-HEK293T) captured at 48 h after infection at indicated multiplicities of infection (MOIs) with AAV2 WT or AAV2.RBDv2 expressing eGFP from an scCMV-eGFP genome. Scale bar, 200 μm. (B) Quantification of the fraction of eGFP-positive cells. Images and quantification were acquired 48 h post infection. Two-way ANOVA, Šídák’s multiple comparisons test: ∗∗∗∗*p* < 0.0001, bars indicating mean ± SEM, *n* = 6 (except for AAV2 WT–ACE2-OE-HEK293T, where *n* = 3).

Next, the transduction efficiency of AAV.RBD vector variants was evaluated in HEK293T cells as well as HEK293T cells stably overexpressing ACE2 (ACE2-OE-HEK293T). SARS-CoV-2 binds ACE2 via its RBD and thereby facilitates cell infection.^24^^,^^25^ We hypothesized that if the RBD of AAV2.RBDv2 is surface exposed and functional, AAV2.RBDv2 should also be able to use that infection mechanism and hence exhibit a higher transduction efficiency in ACE2-OE-HEK293T than in HEK293T cells. As expected, AAV2 WT showed high transduction of HEK293T cells already at the lowest multiplicities of infection (MOIs) of 250 vg/cell, which became saturated at the higher MOIs. The transduction efficiency of the AAV2 WT vector did not change significantly when ACE2 was overexpressed (Figures 3A and 3B). AAV2.RBDv2 vectors showed lower transduction rates compared with AAV2 WT in HEK293T cells at all three MOIs tested, with efficiencies ranging from 27% to 75% (Figures 3A and 3B). However, in contrast to AAV2 WT, the AAV2.RBDv2 vector transduced ACE2-OE-HEK293T cells significantly better than HEK293T cells (Figures 3A and 3B). At 1,000 vg/cell, the transduction efficiency of AAV2.RBDv2 in ACE2-OE-HEK293T cells reached the level of AAV2 WT (98.0 ± 1.3 vs. 99.8 ± 0.2, *p* = 0.956). This ACE2-dependent increase in transduction efficiency could be confirmed in AAV9.RBDv1 (Figure S3). AAV9 generally transduces HEK293T cells with much lower efficiency than AAV2. Nevertheless, we observed significantly higher transduction efficiencies of AAV9.RBDv1 in ACE2-OE-HEK293T cells compared with AAV9 WT (Figure S3). These results suggest that the RBD on the capsid surface of our AAV variants can functionally interact with the ACE2 receptor.

### Immunogenicity of AAV.RBD VLPs in rabbits

**Figure 4:**
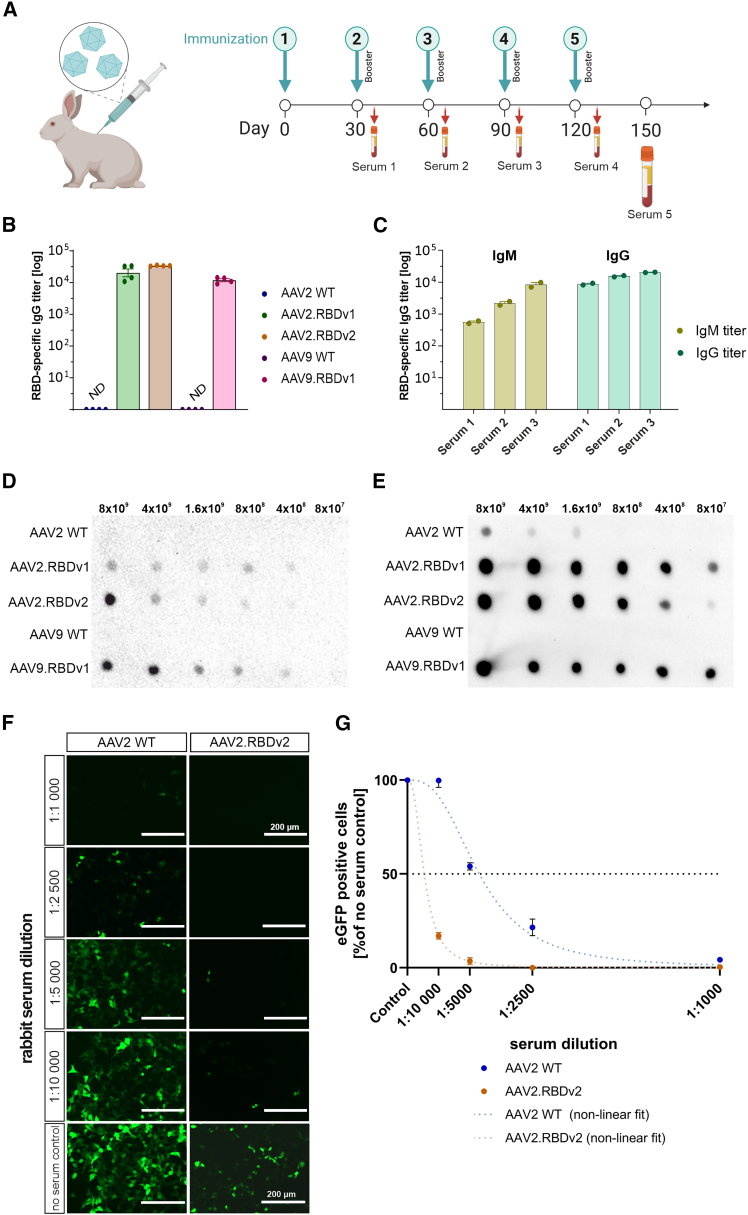
Evaluation of rabbits with AAV.RBD VLPs (A) Schematic representation of the immunization strategy for adult rabbits. AAV.RBD VLPs were administered subcutaneously on day 0 with booster injections every 30 days. Blood was collected 10 days after each booster injection until day 150. Rabbits were immunized with wild-type AAV capsids (AAV2 WT; AAV9 WT) or AAV.RBD capsids (AAV2.RBDv1, AAV2.RBDv2, or AAV9.RBDv1). (B) RBD-specific IgG titers in serum 5 of immunized rabbits tested with a commercially available RBD protein. Bars show mean ± SEM, *n* = 4. (C) IgM (yellow) and IgG (blue) antibody titers in AAV2.RBDv2-immunized rabbit sera 10 days after the first three serum collections. Shown are the endpoint titers of SARS-CoV-2 RBD-specific IgM and IgG antibodies. Bars indicate mean ± SEM, *n* = 2. (D) Evaluation of RBD-binding properties. Dot blot of AAV.RBD and AAV WT capsids immobilized in the indicated total capsid particle amount on a PVDF membrane. The membrane was probed with a commercially available rabbit monoclonal anti-SARS-CoV-2 spike S1 antibody (at 1:500 dilution). Only AAV.RBD capsids show reactivity with the SARS-CoV-2 antibody. (E) Dot blot with AAV.RBD and AAV WT vectors probed with serum of from an AAV9.RBDv1-immunized rabbit (at 1:10,000 dilution). The serum shows a strong reactivity with all AAV.RBD variants. (F) Evaluation of neutralization efficacy of the sera from rabbits immunized with AAV.RBD. Representative fluorescence images of stable ACE2-OE-HEK293T cells that had been transduced with AAV2 WT or AAV2.RBDv2 vectors carrying an scCMV-eGFP genome. Vectors were pre-incubated for 1 h at 37°C at an MOI of 250 vg/cell with indicated dilutions of sera from AAV2.RBDv2-immunized rabbits. Scale bar: 200 µm. (G) Quantification of eGFP-positive cells 48 h post transduction normalized to the respective no serum controls. Non-linear fit of dose vs. normalized response with variable slope. Bars indicate mean ± SEM, *n* = 3.

To evaluate the ability of AAV.RBD to elicit a humoral immune response *in vivo*, we immunized rabbits with AAV.RBD VLPs and AAV WT VLPs, collected serum at different time points for RBD-specific IgG concentrations (Figure 4A). All three AAV.RBD variants induced a strong IgG antibody response with serum titers between 3.9 and 4.5 (values given as 1/log 10 dilution steps), while AAV2 and AAV9 WT capsids did not (Figure 4B). In addition, we examined the antibody response against AAV2.RBDv2 in more detail and compared the RBD-specific IgG and IgM titers (Figure 4C). While the IgG titers had almost reached their maximum after the first booster injection (serum 1), the IgM titers were low at the beginning and increased continuously over the course of immunization with the booster injections (Figure 4C). To confirm the presence of the RBD sequence in AAV.RBD particles, we performed dot blot experiments with AAV2 and AAV9 WT controls and AAV.RBD variants spotted on polyvinylidene difluoride (PVDF) membranes. The membrane was first probed with a commercially available anti-SARS-CoV-2 spike S1-specific antibody. We observed a concentration-dependent RBD-specific signal for all three AAV.RBD variants, but not for the AAV2 and AAV9 WT controls (Figure 4D). This suggested that the RBD sequence was present in all three AAV.RBD variants in a conformation that allows for binding of anti-SARS-CoV-2 spike S1-specific antibody. Next, we probed dot blots spotted with AAV.RBD and AAV WT vectors with serum from rabbits immunized with one of the AAV.RBD variants. Figure 4E shows a dot blot probed at a dilution of 1:10,000 with serum from an AAV9.RBDv1-immunized rabbit. A strong, concentration-dependent signal is seen with all AAV.RBD variants at all dilutions (Figure 4E). Since serotype-independent cross-reactivity was observed, this suggests the presence of antibodies directed against the RBD in this serum. Similar results were obtained with sera from rabbits immunized with AAV2.RBDv1 (Figure S2A) and AAV2.RBDv2 (Figure S2B).

After detecting the presence of anti-RBD antibodies with high binding affinity in sera from rabbits immunized with AAV.RBD, we subsequently investigated the presence of NAbs. To this end, we used the previously introduced ACE2-OE-HEK293T cell line to compare the transduction efficiency of AAV2.RBDv2 and AAV2 WT vectors that have been pre-incubated with anti-AAV2.RBDv2 serum at four different dilutions (Figures 4F and 4G). At the highest serum concentration (1:1,000 dilution), both vectors, AAV2 WT and AAV2.RBDv2, were neutralized by over 95%, suggesting the presence of antibodies directed against the AAV capsid part in the anti-AAV2.RBDv2 serum (Figure 4F, top row). However, transduction of AAV2.RBDv2 was more strongly inhibited by the serum than transduction of AAV2 WT, showing an IC_50_ of 0.023% (95% confidence interval [CI], 0.021%–0.025%) compared with an IC_50_ of 0.0055% for AAV2 WT (95% CI, 0.0045%–0.0062%) (Figure 4G). This corresponds to an approximately 4-fold stronger inhibition of AAV2.RBDv2 compared with AAV2 WT, indicating the presence of high levels of RBD-specific NAbs in the serum of AAV2.RBDv2-immunized rabbits.

### AAV.RBD vectors are neutralized by plasma of individuals vaccinated with Comirnaty

**Figure 5:**
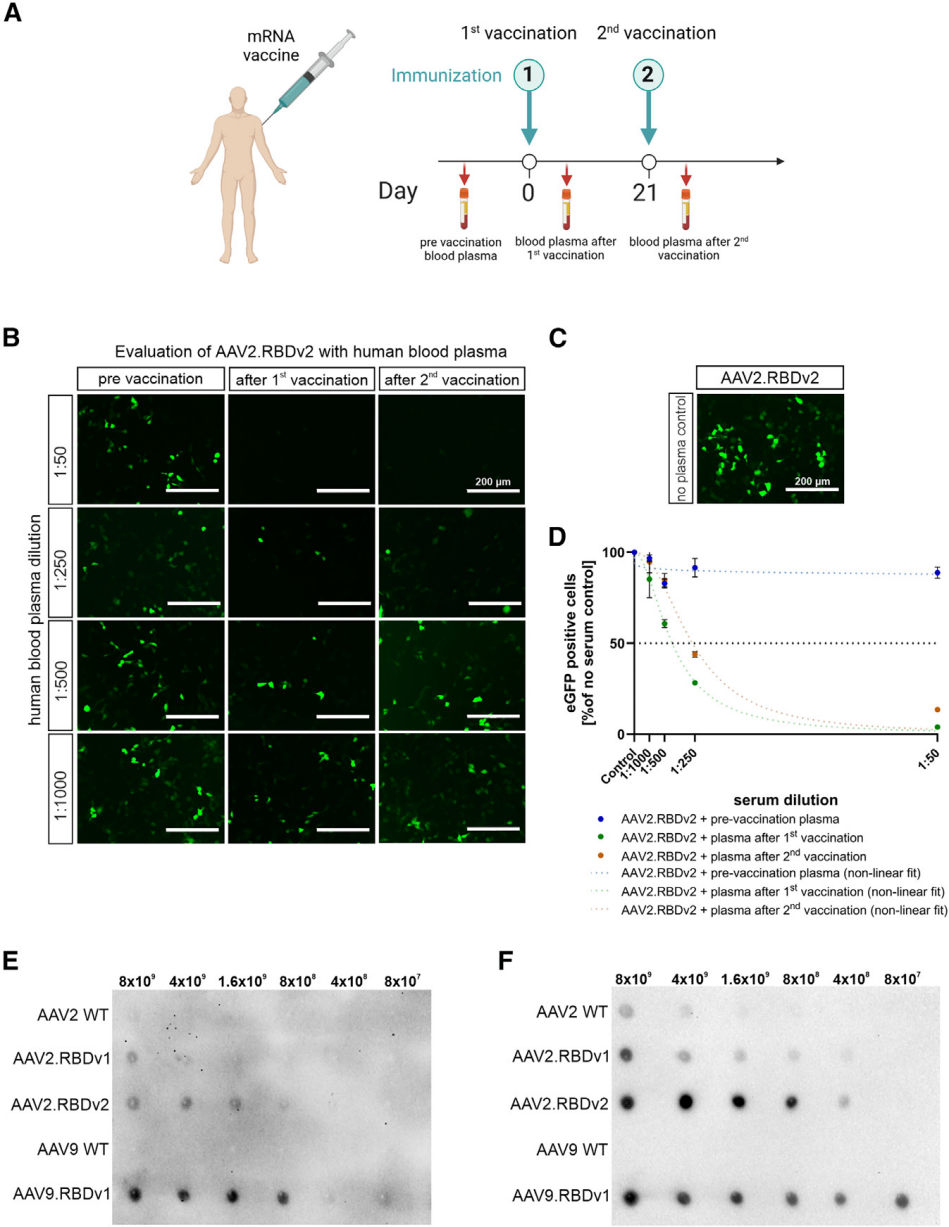
Affinity and neutralization capacity of human anti-SARS-CoV antibodies on AAV.RBD variants (A) Vaccination schedule of blood plasma donors as carried out at the beginning of 2021 in accordance with the STIKO recommendation in force at that time with Comirnaty mRNA-based vaccine against the SARS-CoV-2 spike protein. Pre-vaccination plasma was collected before vaccination, plasma 1 was collected 2 weeks after the first vaccination, and plasma 2 was collected 1 week after the second vaccination. (B) Neutralization of AAV2.RBDv2 by human plasma obtained after vaccination. Representative fluorescence images showing transduction of AAV2.RBDv2-expressing eGFP in stable ACE2-overexpressing HEK293T cells at an MOI of 250 vg/cell. Vectors were pre-incubated for 1 h at 37°C with four different dilutions (1:50, 1:250, 1:500, 1:1,000) of human plasma from individuals that had been vaccinated with Comirnaty. Epifluorescence images were acquired 48 h post infection. Scale bar: 200 μm. (C) Control transduction in the absence of plasma (no plasma control). Scale bar: 200 μm. (D) Quantification of eGFP-positive cells 48 h post transduction normalized to the respective controls without plasma. Non-linear fit of dose vs. normalized response with variable slope. Data are plotted as mean ± SEM, *n* = 3. (E) Dot blot of AAV.RBD and AAV WT vectors after incubation with human plasma 1 after Comirnaty vaccination (at 1:500 dilution). AAV.RBD and AAV WT capsid variants were spotted on the PVDF membrane at the indicated total capsid particle amount. (F) The membrane from (E) was stripped and re-probed with AAV9.RBDv1-immunized rabbit serum at a 1:10,000 dilution.

Next, we wanted to investigate whether human anti-SARS-CoV-2 spike antibodies could cross-react with AAV.RBD. Therefore, we performed similar dot blot and neutralization assays, this time using plasma from individuals who had been vaccinated with the mRNA-based vaccine Comirnaty and in whom the presence of anti-SARS-CoV-2 spike IgG antibodies had been confirmed by ELISA (see materials and methods for more details). Plasma and peripheral blood monocyte cell (PBMC) samples from three individual donors obtained before vaccination, and after the first and second Comirnaty dose were used for the experiments (Figure 5A). AAV2.RBDv2 and AAV2 WT vectors were pre-incubated with four different dilutions of donor plasma (1:50, 1:250, 1:500 and 1:1,000) prior to transduction of ACE2-OE-HEK293T cells. All dilutions of plasma collected before vaccination showed transduction levels similar to those of the plasma-free control, indicating the absence of NAbs (Figures 5B–5D). In contrast, plasma samples obtained after the first and the second vaccination showed efficient neutralization of AAV2.RBDv2 (Figures 5B–5D). More specifically, the plasma obtained after the first vaccination inhibited transduction with an IC_50_ of 0.0025% serum (95% CI, 0.0022%–0.0029%) and the plasma after the second vaccination inhibited transduction with an IC_50_ of 0.0038% serum (95% CI, 0.0033%–0.0050%) (Figure 5D). Thus, AAV.RBDv2 can be efficiently neutralized by anti-SARS-CoV-2 spike antibodies present in plasma from mRNA (Comirnaty)-immunized individuals, thereby confirming exposure of the SARS-CoV-2 RBD epitope at the capsid surface of AAV.RBD in a similar way as the RBD in the proteins translated from the mRNA vaccine. Binding of antibodies from plasma from mRNA-vaccinated individuals was demonstrated by probing dot blots with spotted AAV.RBD. As shown in Figure 5E, human antibodies cross-reacted with all AAV.RBD variants, but not with AAV2 or AAV9 WT controls, supporting the antibody specificity to the AAV.RBD epitopes (Figure 5E). The dot blot was then stripped and re-incubated with serum from AAV9.RBDv1-immunized rabbits confirming proper loading (Figure 5F). These results demonstrate the similarity of the SARS-CoV2 spike protein produced in human mRNA vaccines with the RBD displayed by the engineered AAV capsids.

### AAV.RBD-specific cellular immune responses of individuals vaccinated with Comirnaty

**Figure 6:**
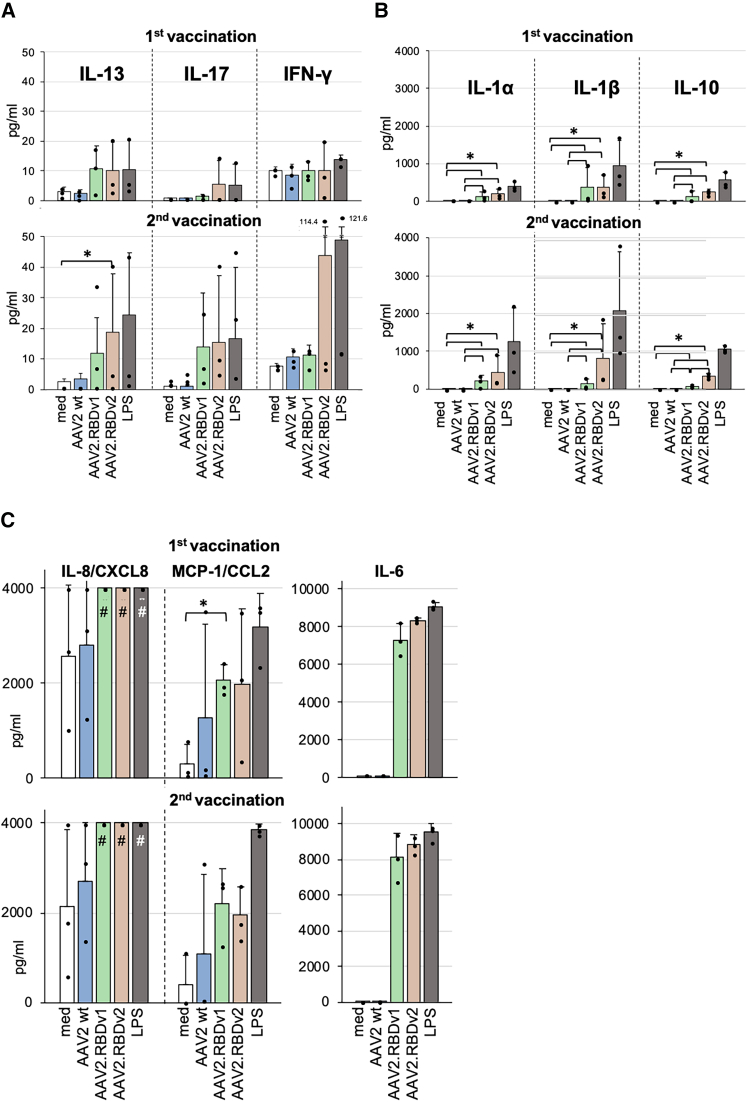
*In vitro* cytokine responses of human PBMC to AAV2 WT and AAV.RBD variants (A–C) Culture supernatants of human cells were stimulated with empty capsids and LPS as positive control or medium only as described and indicated. The culture supernatants were analyzed for the secretion of the following cytokines and chemokines: (A) IL-13, IL-17, and IFN-γ; (B) IL-1α, IL-1β, and IL-10; and (C) IL-8/CXCL8, MCP-2/CCL2, and IL-6. Data shown are means +SD from three donors (same as serum donors). Brackets and asterisks mark significant differences with *p* ≤ 0.05; #values above the detection limit of the assay.

**Figure 7:**
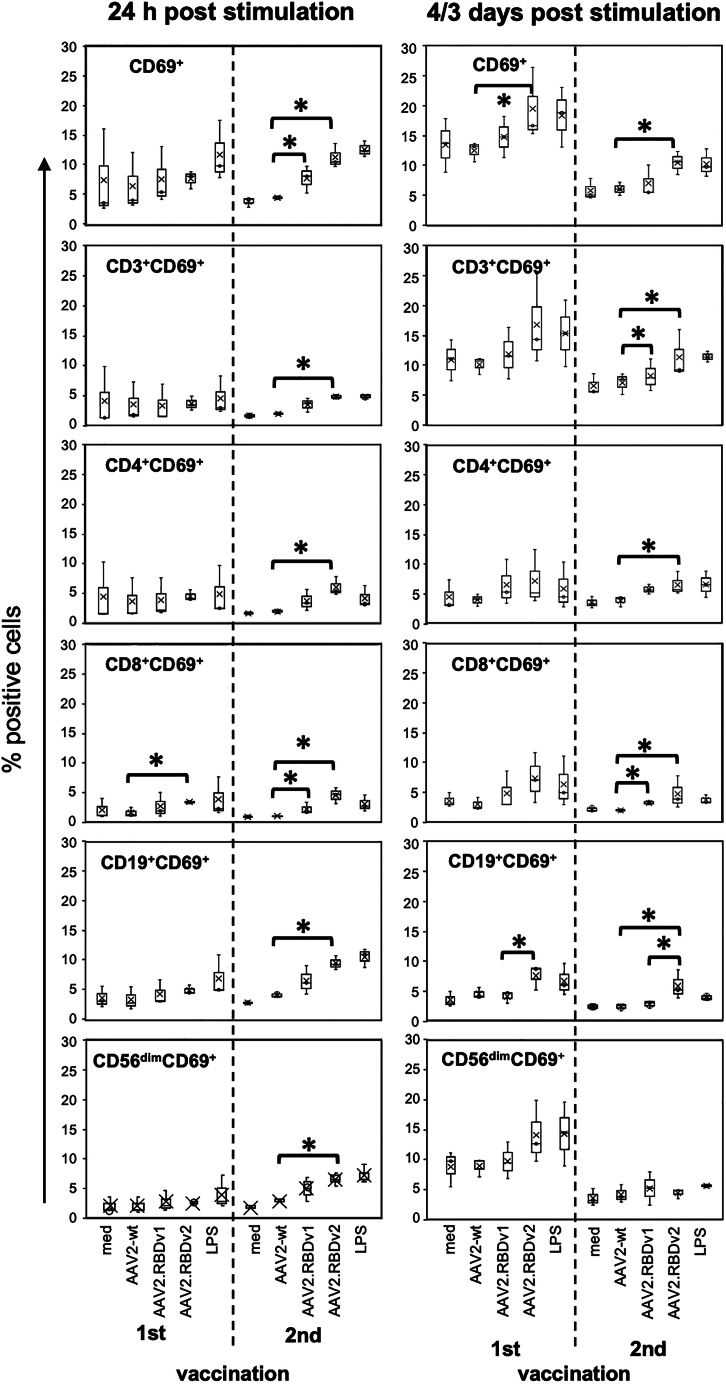
Proliferation of activated PBMC populations in response to AAV2 WT and AAV.RBD variants Peripheral lymphocytes from the same donors as in Figure 6 (*n* = 3) were stimulated and stained as described. Total lymphocytes were gated according to FCS/SSC and percentage of activated (CD69+) cells and cells co-expressing CD3+, CD4+, CD8+, CD19+, or CD56dim+ are shown after 24 h stimulation (left panel) and after 4 days (after first vaccination) or 3 days (after second vaccination) stimulation as indicated (right panel). The central line within the bars shows the median, the x represents the mean and the whiskers above and below display the minimum and maximum within 1 interquartile range of the lower and upper quartile. Brackets and asterisks mark significances with *p* ≤ 0.05.

To investigate how AAV.RBD affects cytokine and chemokine secretion from PBMCs of individuals who had been mRNA vaccinated, we analyzed cellular responses of the plasma donors after *in vitro* stimulation with AAV2 WT, AAV2.RBDv1, and AAV2.RBDv2. PBMCs stimulated with lipopolysaccharide (LPS) served as positive control and unstimulated PBMCs (only medium) as negative control. After stimulation, culture supernatants were collected daily and pooled to detect cytokine production across a range of time points. The secretion of the T cell-specific cytokines interleukin-13 (IL-13), IL-17, and interferon-gamma (IFN-γ) in response to AAV2.RBD variants was highly variable among the three donors but generally in a range similar to LPS. These cytokines were found increased in the cultures from the youngest donor (donor 2). After the second vaccination, the IFN-γ response of this donor was particularly strong against AAV2.RBDv2 and LPS (Figure 6A). IL-10, a regulatory cytokine also produced by T helper cells, and the two monocyte-derived cytokines (monokines) IL-1α and IL-1β were secreted by cells from all donors at significantly higher levels in response to AAV2.RBDs compared with AAV2 WT, especially after the second immunization (Figure 6B). A very high and persistent secretion of IL-6 was observed in response to both AAV2.RBD variants and to LPS, but not to AAV2 WT (Figure 6C). The secretion of IL-8/CXCL8 after stimulation with AAV2.RBD variants or LPS was massively increased to levels exceeding the test range, therefore no significance could be calculated (Figure 6C). MCP-1/CCL2 responses to AAV2.RBD variants were only slightly enhanced compared with AAV2 WT. The weak or lack of recognition of AAV2 WT capsids is in accordance with the humoral data described above (Figure 5). In support of the view that AAV2.RBDs may induce a stronger cytokine/chemokine response than AAV2 WT, we also observed increased proliferation of lymphocyte populations to AAV2.RBD variants (Figure 7). Increased proliferation was observed after the first and second vaccination for B (CD19+CD69+), helper (CD4+), and cytotoxic (CD8+) T cell populations as well as for CD56^dim^ NK cells, which are specialized in the recognition of and defense against viruses. Generally, these effects were most consistently seen in response to AAV2.RBDv2 (Figure 7).

### AAV2.RBD VLPs reactivate SARS-CoV-2-specific memory T cell responses

**Figure 8:**
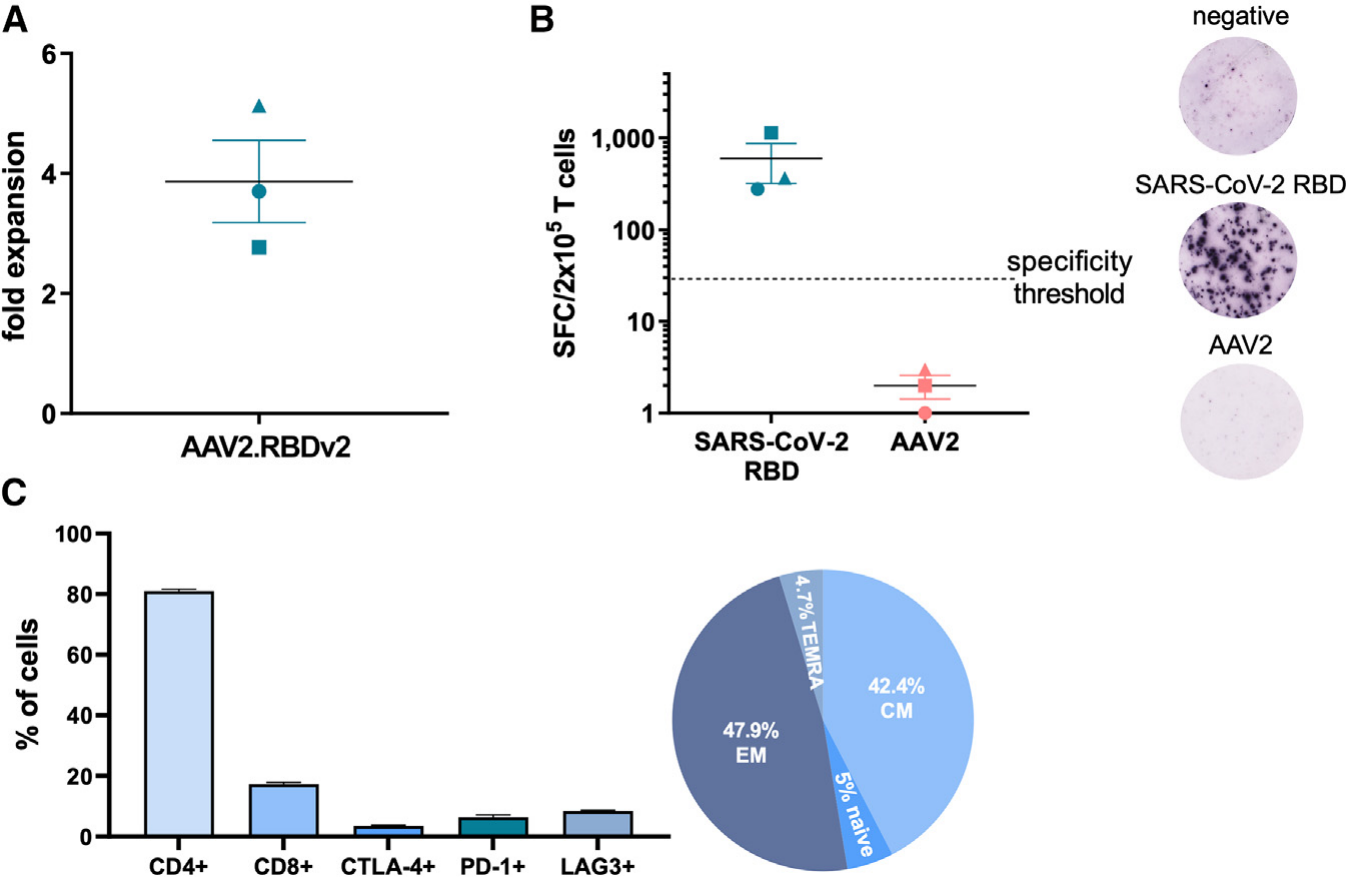
Memory-specific T cell response against SARS-CoV-2 after AAV2.RBDv2 pulsing (A) Fold expansion of T cells after pulsing with AAV2.RBDv2 and culturing for 10 days. Each dot represents a single donor. (B) IFN-γ SFCs upon stimulation of AAV2.RBDv2-stimulated T cells with either SARS-CoV-2 spike RBD or AAV2 pepmixes. Each dot represents a single donor. The dotted line illustrates the specificity threshold. Representative ELISpot of a donor’s T cell response either unpulsed or after pulsing with SARS-CoV-2 spike RBD or AAV2 pepmixes (100,000 T cells per well were plated). (C) Immunophenotype of AAV2.RBDv2-stimulated T cells. Data shown are means ± SEM. CM, central memory; EM, effector memory; TEMRA, terminally differentiated effector memory cells re-expressing CD45RA.

Next, we went on to characterize antigen-specific T cell responses to AAV2.RBDv2. To this end, PBMCs from three healthy donors—previously vaccinated with Comirnaty and tested positive for SARS-CoV-2 infection (median weeks after vaccination: 79 [range 33–83] and after infection: 13 [range 8–34])—were stimulated *in vitro* with AAV2.RBDv2-pulsed antigen-presenting cells (APCs) and assessed for a specific immune response after a 10-day culture. To ensure that AAV2.RBDv2 could adequately present SARS-CoV2-RBD epitopes for T cell recognition, a dual T cell stimulation approach was employed. First, PBMCs from one donor were directly treated with AAV2.RBDv2 VLPs to generate pathogen-specific T cells, according to an established protocol that uses overlapping peptide pools.^26^^,^^27^^,^^28^^,^^29^ This approach allowed AAV2.RBDv2 VLPs to directly pulse APCs within the PBMC mix, which subsequently stimulated the T cells. Concurrently, an indirect approach involved pre-pulsing a fraction of PBMCs, serving as APCs, with AAV2.RBDv2 VLPs overnight, before co-culture with the remaining fraction of PBMCs containing T cells at 1:1 or 1:2 ratios. This pre-pulsing step aimed to induce antigen saturation of the APCs, thus optimizing subsequent T cell activation. Both, direct exposure and pre-pulsing yielded an average 3.9 ± 0.7-fold expansion of cultured cells (Figure 8A). Notably, in both strategies, AAV2.RBDv2-stimulated T cells from vaccinated and SARS-CoV2-infected donors were shown to have strong specificity against SARS-CoV-2 RBD antigen, without cross-reactivity with the AAV2 viral protein, which forms the backbone of AAV2.RBDv2 (Figure 8B). This argues that AAV2.RBDv2 VLPs effectively elicit robust antigen-specific T cell responses, specifically targeting the intended antigen and not AAV antigens. In addition, T cells primed with AAV2.RBDv2 were predominantly CD4^+^ (as expected with the SARS-COV-2 cellular immune responses) but also included CD8^+^ T cells, presenting a non-exhausted profile with over 90% expressing central and effector memory markers (Figure 8C).

## Discussion

Although AAVs have a low immunogenic profile compared with other viruses, such as adenoviruses, they have already been extensively explored as vaccines.^30^^,^^31^ In most cases, AAVs have been used as vectors for the expression of immunogenic antigens to ensure long-lasting protection, e.g., against dengue virus, herpes simplex virus 2, human immunodeficiency virus 1, human parvovirus 16, or influenza A virus.^32^^,^^33^^,^^34^^,^^35^^,^^36^^,^^37^ This concept was also intensively pursued during the COVID-19 pandemic and several vaccination approaches against SARS-CoV-2 with AAV vectors of different serotypes (e.g., AAV5, AAV9, AAVRh32.33) have been developed, which either expressed the entire S1 subunit^6^^,^^38^ or parts of the RBD.^39^^,^^40^^,^^41^ The aim was that the AAV vector technology could provide a widely applicable vaccine due to its well-established production process and relatively good stability at 4°C or even at room temperature.^6^ Despite promising results, none of the AAV-based vaccines have yet received marketing authorization.

The ease of genetic manipulation of the AAV capsid^42^ offers additional opportunities to develop AAV variants that present immunogenic peptides to induce an immediate humoral response against the immunogenic sequence.^43^^,^^44^ In one strategy, short amino acid sequences are incorporated into one or both surface-exposed loops of the AAV capsid.^43^ Another strategy involves fusing immunogenic sequences to the N terminus of VP2, which is then presented at the capsid surface.^44^ When such engineered AAV capsids are combined with a vector genome expressing an antigenic sequence, this technology enables a second delayed exposure to the antigen following the immediate humoral response in a “one-shot prime boost” regimen.

In this study, we further expanded the scope of AAV as an immunogenic scaffold by introducing large immunogenic peptide insertions into the surface-exposed variable loop region VIII of AAV2 and AAV9 viral proteins. In this way, the immunogenic sequence is inserted in all three VPs and presented 60 times on the AAV capsid surface. Given its proven immunogenicity,^17^^,^^19^^,^^20^^,^^21^ we used the RBD of SARS-CoV2 as a model sequence and generated three different AAV.RBD capsids. We could show that the AAV2.RBD and AAV9.RBD capsids can be used to produce VLPs and vectors with AAV features, but also with SARS-CoV-2 properties conferred by the inserted sequence. In affinity chromatography using AAVx columns AAV2.RBD and AAV9.RBD VLPs and vectors showed binding and elution profiles comparable with AAV2. AAVx columns are coated with several anti-AAV capsid antibodies directed against specific conformational epitopes of different AAV serotypes, such as AAV2 or AAV9. The behavior of AAV.RBD on the AAVx column thus confirmed the formation of capsids with AAV properties and suggested that the complex process of AAV capsid assembly^45^ was not impaired by the large insertion.

When produced as vectors with genome, all three AAV.RBD variants resulted in poor genome titers that were two to three log units lower than the corresponding WT vectors. However, the capsid titers were only one log unit lower than those of the corresponding WT vectors. This suggests that AAV.RBD form multimeric capsids that may be leaky and/or have low packaging efficiency, as observed in the context of thermal stress, such as repeated freeze/thaw cycles^46^ or elevated temperatures.^47^ In addition, differences in capsid modifications or steric hindrances around the pore at the 5-fold symmetry axis, which is the entry point for the vector genome into the assembled AAV capsid, could lead to low genome titers.^48^ While the only one log unit reduced capsid titers might be sufficiently high for generating vaccines of highly immunogenic sequences, further optimization to enhance the production yield is warranted. In particular, if someone is aiming to combine using this capsid technology with a vector genome, e.g., in a one-shot prime boost approach.^44^ Possible optimizations could include the production of AAV.RBD as mosaic vectors with WT capsid at different mutant/WT ratios.^49^

Despite the relatively low packaging efficiency, the AAV.RBD vectors were still able to transduce cells, although with significantly lower efficiency (see Figures 3A and 3B). Both the AAV2.RBDv2 and the AAV9.RBDv1 vectors were able to transduce HEK293T cells, albeit with significantly lower efficiency then the respective parental WT AAV. Importantly, their transduction efficiency was significantly increased in HEK293T cells overexpressing the ACE2 receptor. This indirectly confirms that AAV.RBD vectors present the RBD of SARS-CoV-2 in a functional conformation that allows efficient binding to the native ACE2 receptor to mediate enhanced cell infection and transduction. Further evidence that the RBD is present in a native conformation on the capsid surface is the binding of antigen-specific NAbs from the plasma of individuals vaccinated with the SARS-CoV2 mRNA vaccine Comirnaty. This proper presentation of the SARS-CoV-2 RBD might have contributed to the strong humoral response elicited by all three AAV.RBDs in rabbits. Of note, all sera obtained from rabbits immunized with one of the three AAV.RBDs showed a strong cross-reactivity with the other two AAV.RBDs but bound only weakly or not at all to the parental WT AAV. In addition to inducing antibodies with high affinity for the target antigen, AAV.RBD also induced antibodies with high neutralization activity, as sera from immunized rabbits reduced the transduction efficiency of AAV.RBD vectors in ACE2-OE-HEK293T cells when pre-incubated with the vectors.

We also investigated the secretion of cytokines and chemokines and the proliferation of activated lymphocytes after AAV.RBD stimulation of human PBMCs derived from donors vaccinated with Comirnaty. We observed a slightly increased secretion of very low amounts of T cell cytokines, especially the Th1 marker cytokine IFN-γ as typical antiviral response, IL-17 from Th17 cells, and IL-13, secreted by Th2 cells, NKT cells, and some innate cell types, and suggested to be protective in SARS-CoV-2 infection.^50^ We also observed an increase of IL-10 in response to AAV.RBD, which is anti-inflammatory but also promotes B cell and antibody responses. In response to AAV.RBD, we also found a specific upregulation of monocyte-produced inflammatory cytokines and chemokines, such as IL-6, indicating a concerted immune activation comprising cells of the innate and adaptive immune response. We also observed increased proliferation of activated (CD69^+^) T and B and CD56^dim^ NK cell populations in response to AAV.RBD, which markedly increased after the booster vaccination compared with the first immunization, especially to AAV2.RBDv2. Responses from both T helper (CD4^+^) as well as cytotoxic T cells (CD8^+^) were obtained.

To better characterize the potential of AAV.RBD as a vaccine candidate, we stimulated *ex vivo* PBMCs from donors with hybrid immunity against SARS-CoV-2 using AAV.RBD and tested the robustness and specificity of the resulting T cell responses. Upon stimulation, we observed significant expansion of T cells with a strong specificity for the SARS-CoV-2 RBD antigen without cross-reactivity to unrelated (AAV2 viral protein) antigens, highlighting the precision of AAV.RBDs in eliciting the desired immune response. Notably, the absence of cross-reactivity with AAV antigens further underscores the specificity of the immune response to SARS-CoV-2 RBD, which is a crucial factor for vaccine safety and efficacy. Furthermore, the presence of both CD4^+^ and CD8^+^ T cells, exhibiting non-exhausted phenotypes and high levels of memory markers, suggests the potential of AAV.RBDs to reactivate a durable immune response, which is essential for long-term protection.^51^ This is particularly important in the context of pandemic threats, such as SARS-CoV-2, where the durability of protection and the ability to respond to future exposures are critical.

In summary, we have introduced a novel next-generation vaccine platform that uses modified AAV capsids as a scaffold to present large immunogenic sequences to the host immune system. Our findings demonstrate that this technology enables (1) a strong and specific immune response against the inserted antigen and (2) at the same time only a mild response to the “carrier” AAV capsid. Therefore, the AAV capsids we have engineered appear to be suitable scaffolds for the presentation of large immunogenic sequences, eliciting a strong and specific immune response. This technology holds promise as a versatile vaccine platform for combating various infectious pathogens.

## Materials and methods

### Cloning of modified AAV capsids

Three capsid variants were generated on the basis of AAV2 or AAV9 using Gibson assembly.^52^ SARS-CoV-2 RBD coding sequences corresponding to amino acids 333–529 (referred to as variant S1.1) or 300–505 (variant S1.2) (GenBank: QHD43416) (Table S1) were cloned with three preceding and two following alanines as linkers in Cap from pRC'99^53^^,^^54^ at position N587 and pAAV2/9\-sw-SEED at position Q588 for AAV2 and AAV9, respectively. The S1.1 insertion was generated in both AAV2 and AAV9 contexts, while S1.2 was generated with AAV2 only. The resulting plasmids were named HtW2_S1.1 (for AAV2.RBDv1), HtW2_S1.2 (for AAV2.RBDv2), and HtW9_S1.1 (for AAV9.RBDv1).

### Production and purification of AAV vectors

Production of AAV vectors was performed via triple transfection of pAdHelper, pRep/Cap, and pTransgene (pAAV2.1_scCMV-eGFP^55^) plasmid into HEK293T cells as described previously.^56^ Genome-free (“empty capsids”) VLPs were produced via double transfection with the pAdHelper and pRep/Cap plasmids, omitting the pTransgene plasmid.

For *in vitro* experiments, vector purification was performed with discontinuous iodixanol gradient ultracentrifugation as described previously followed by liquid chromatography.^56^ Apart from the discontinuous iodixanol gradient ultracentrifugation no other full/empty enrichment step was carried out. AAV.RBD capsid binding properties were analyzed by affinity chromatography using a Poros capture select AAVx column (POROS GoPure AAVX Pre-packed Column, ThermoFisher Scientific). *In vitro* characterization was performed using anion exchange chromatography with a HiTrap Q FF Sepharose HPLC column (17-5156-01, Cytiva) and the PrimeView 5.31 software (GE Healthcare). VLPs for immunization of rabbits were purified via cation exchange chromatography with the CIMmultus SO_3_ column (BIA separations/Sartorius). Following HPLC purification, samples underwent a final concentration step using Amicon Ultra-4, PLHK Ultracel-PL membrane filters (Sigma-Aldrich). Genomic titers were determined using RT-qPCR^56^ and protein (capsid) titers were determined by AAV2 ELISA (PROGEN Biotechnik) or UV-vis spectroscopy, dynamic light scattering, and SLS (Stunner Apparatus, Unchained Labs).

### Modeling of AAV capsids

*In silico* modeling of VP3 monomers was performed with AlphaFold2 (Google DeepMind, ColabFold).^57^ Default parameters were used, and the top-scoring model was accepted for further analysis. Models were visualized using the python-based Chimera software (Chimera 14.1, UCSF).^58^

### Generation of ACE2-overexpressing HEK293T cells

To generate ACE2-OE-HEK293T, the ACE2 sequence was amplified from pCEP4-myc-ACE2 (no. 141185, Addgene) and cloned under control of a CMV promoter into a piggyBac vector with hygromycin B resistance gene (System Biosciences). The resulting plasmid, PB_CMV-ACE2-SV40pA_MCS/EF1-Hygro, was transfected into HEK293T cells (Clontech) using Xfect Transfection Reagent (Takara) to generate the ACE2-overexpressing HEK293T cells (ACE2-OE-HEK293T). After negative selection with hygromycin B (1 mg/mL, ThermoFisher Scientific) single-cell clones were transferred into a 96-well plate with fresh medium and further cultivated. ACE2 expression was assessed using quantitative real-time qPCR. The clone with the highest gene expression was used for further experiments.

### *In vitro* transduction assays

Transduction efficiency of AAV vectors was assessed in HEK293T cells and ACE2-OE-HEK293T cells cultured in 4.5 g/L high-glucose Dulbecco’s modified Eagle’s medium (DMEM) + GlutaMAX (ThermoFischer Scientific), supplemented with 10% fetal bovine serum (FBS) and 1% penicillin/streptomycin. Fluorescence images were collected 48 h after infection with the EVOS fluorescence microscope (EVOS FL Cell Imaging System, ThermoFisher Scientific). Quantification of GFP-positive cells was conducted after 48 h with the Countess II FL Automated Cell Counter (ThermoFisher Scientific). MOIs have been calculated based on the vector genome titer (vg/μL). For neutralization assays, vectors were prediluted in fresh medium at an MOI of 250 vg/cell and incubated with serum of immunized rabbits or blood plasma of human donors vaccinated with Comirnaty at various dilutions for 1 h at 37°C. A non-transfected control without blood plasma was treated equally. Culture medium was replaced by the AAV-serum mixture and incubated for 48 h. Transduction efficiency was evaluated as described above. To calculate the IC_50_ values, a non-linear fit with variable slope was calculated with GraphPad Prism 9.1 (Graph-Pad, San Diego), based on the formula Y = 100/(1 + (IC_50_/X)^HillSlope^), where Y is the transduction efficiency in percent and X is the serum concentration in percent.

### Immunization of rabbits

All procedures were carried out in accordance with German law for animal protection and with the European Directive, 2010/63/EU, and were approved by the government of Upper Bavaria (Munich, Germany, license no. 55.2-2532.Vet_03-17-110). In brief, two female ZIKA hybrid rabbits (3 months; 2.8 kg body weight) were immunized using standard procedures with VLPs each of AAV2 WT, AAV9 WT, AAV2.RBDv1, AAV2.RBDv2, and AAV9.RBDv1. Primary immunizations were with 100 μg VLPs emulsified in Freund’s complete adjuvant (Sigma-Aldrich). Booster injections were with 50 μg of VLPs at 4-week intervals with Freund’s incomplete adjuvant (Sigma-Aldrich). Blood samples were taken 10 days after the first, second, and third booster injection. Blood was allowed to clot overnight at 4°C before sera were gained by centrifugation at 1,200 × *g* for 20 min at 4°C and stored in aliquots at −20°C until use. Preimmune sera (5 mL) were taken 1 week before starting with immunizations. Final blood collection was performed on day 150 by cardiocentesis under general anesthesia.

Serum of immunized rabbits was evaluated for RBD-specific IgM and IgG antibodies using a custom-made ELISA. 96-well plates were coated with 100 ng of SARS-Cov-2 WT RBD (no. SPD-C52H2, Acro Biosystems) in carbonate buffer for 1 h (0.2 M sodium carbonate [pH 9.4]), subsequently blocked with 3% (m/v) sodium caseinate (Sigma-Aldrich) in phosphate-buffered saline (PBS) (pH 7.4) for 30 min, washed, and – 3 log dilutions of the antisera in PBS containing 0.1% (m/v) sodium caseinate were applied to the plates. The plates were incubated with the sera for 2 h at room temperature, washed, and subsequently incubated for 1 h with horseradish peroxidase-labeled goat anti-rabbit IgG (no. ab6721, Abcam) and goat anti-rabbit IgM (no. ab9167, Abcam), each diluted 1:5,000 in PBS for 30 min. After final washes, antibody binding was visualized with TMB substrate solution (no. N301, Sigma-Aldrich) and measured with an EPOCH/2 plate reader (BioTek) at λ = 450 nM. Assays were performed in quadruplicates and titers were defined as the dilution showing a signal above 2 standard deviations (SD) above background.

### Dot blot assay

VLPs were applied directly on a methanol-activated PVDF membrane (Roth) and probed with the following sera or primary antibodies: rabbit anti-SARS-CoV-2/α-RBD antibody (1:500 dilution, Sino Biological); rabbit anti-AV2.RBDv1, anti-AAV2.RBDv2, anti-AAV9.RBDv1, anti-AAV2, and anti-AAV9 sera (all 1:10,000; LMU Department of Veterinary Sciences); and human anti-SARS-CoV-2 spike plasma (from donors 1, 2, and 3; see Table S2; all 1:500). Plasma was collected from three healthy donors with signed informed consent and approved by the ethics committee of the University Hospital of the LMU Munich before, 2 weeks after the first and 1 week after the second vaccination with Comirnaty (BNT162b2, BioNTech/Pfizer). None of the donors had been infected with SARS-CoV2 before the blood samples were taken. Secondary detection was performed using goat anti-human IgG (ThermoFischer Scientific), rabbit anti-human IgM (Abcam), or donkey anti rabbit IgG (GE Healthcare).

### PBMC and human plasma isolation

This procedure refers to experiments shown in Figures 5, 6, and 7. The study was approved by the ethics committee of the University Hospital of the LMU Munich. Peripheral blood was collected from healthy, unexposed donors 1, 2, and 3 two weeks after the first and one week after the second vaccination with Comirnaty (Table S2). PBMCs were isolated from heparinized blood by Ficoll density gradient centrifugation and immediately cultured for the proliferation assays with subsequent FACS analysis and cytokine bioplex assay (as detailed below). PBMC culture was done in DMEM/high-glucose medium supplemented with 2 mM L-glutamine, 0.27 mM asparagine, 100 U/mL penicillin/100 μg/mL streptomycin, 1% non-essential amino acids, 1 mM Na-pyruvate (all from Sigma-Aldrich), and 5% serum replacement Panexin CD (PAN-Biotech) at 37°C/7% CO_2_ and stimulated as described below. Plasma was collected from the same donors at the same time and immediately frozen and stored at −20°C until used for antibody determination and transduction inhibition experiments. SARS-CoV2-spike-specific antibodies in human plasma were determined using the Abbott Architect SARS-CoV-2 IgG II Quant chemiluminescent microparticle immunoassay (Abbott) to detect IgG antibodies to the receptor binding domain/S1 subunit of the SARS-CoV-2 spike. The analysis was performed according to the manufacturer’s instructions on the Architect i1000SR Immunoassay Analyzer (Abbot) in the accredited routine diagnostic laboratories of the Max von Pettenkofer Institute (LMU Munich).

For stimulation of PBMCs, determination of lymphocyte subtypes and cytokine bioplex assay (Figures 6 and 7), freshly isolated PBMCs (2–5 × 10^6^/mL) were stimulated with LPS (tlrl-eblps, InvivoGen), AAV2 WT, AAV2.RBDv1, and AAV2.RBDv2 and subsequently cultured as described above. AAV concentrations were 1 × 10^9^ vg/mL/well ≈ 6 × 10^9^ capsids/well. Medium and AAV2 WT were used as negative controls to determine baseline activation, and 5 μg/mL LPS as a positive control for maximal stimulation. The stimulation was performed for 24 h and 4 days after the first vaccination or for 24 h and 3 days (instead of 4 days; this has proven to be optimal for lymphocytes pre-activated *in vivo* by the booster vaccination) after the second vaccination. Supernatants from triplicate cultures were collected at three time points after the first (24, 72, and 96 h) and second vaccination (24, 48, and 72 h) and stored at −80°C. Equal volumes of supernatants from each stimulation were pooled and IL-1β, IL-1ra, IL-6, IL-8, IL-10, IL-12p40, IL-12p70, IL-13, IL-17, IFN-γ, MCP-1, MCP-3, PDGF-BB, and VEGF-A measured using a cytokine/chemokine multiplex assay kit (Bio-Rad) according to the manufacturer’s instruction. Data were acquired with a Bio-Plex Luminex Reader and analyzed with Bio-Plex Manager software (Bio-Rad). The final values obtained in the bioplex analysis were calculated from the median value of fluorescence of at least 50 measured beads per analyte and sample. Only those cytokines/chemokines detected in the culture supernatants are shown.

### T cell stimulation assay

This procedure refers to experiments shown in Figure 8. The study was approved by the Institutional Review Board of the George Papanikolaou Hospital (Thessaloniki, Greece). Under signed informed consent, peripheral blood was collected from healthy donors 4, 5, and 6 (Table S2), previously vaccinated with Comirnaty (BNT162b2, BioNTech/Pfizer) and tested positive for SARS-CoV-2 infection, to investigate the induction of a T cell immune response by genome-free “empty” AAV.RBD VLPs. PBMCs were isolated from heparinized blood samples using Ficoll density gradient centrifugation. In the direct stimulation approach, isolated PBMCs were pulsed with 2.5 × 10^9^ capsids/well AAV2.RBDv2 VLPs and cultured in T cell medium (Advanced RPMI 1640 supplemented with 45% Click’s medium, 2 mM GlutaMAX, and 10% FBS) supplemented with IL-7 and IL-4. In an indirect stimulation approach, a fraction of PBMCs was pre-pulsed with 2.5 × 10^9^ capsids/well AAV2.RBDv3 VLPs overnight and the next day were washed and co-cultured with the remaining fraction of PBMCs containing T cells at 1:1 or 1:2 ratios in T cell medium supplemented with IL-7 and IL4. Every 2–3 days, cultures were replenished with fresh medium. Cultures were terminated on days 9–11. Each donor sample was tested in duplicates.

### Enzyme-linked immunospot assay

For experiments shown in Figure 8, AAV.RBD VLP-stimulated T cells were pulsed with either SARS-CoV-2 spike RBD or AAV2 pepmixes (jpt peptide technologies) and the secretion of IFN-γ by the stimulated cells was measured by enzyme-linked immunospot (Elispot) (Mabtech). Spot-forming cells (SFCs) were counted on Eli.Scan Elispot scanner (A.EL.VIS) using Eli.Analyze software V6.2.SFC. The specificity of cells was expressed as SFCs per input cell numbers after subtracting the background spots (unstimulated cells). Response was considered positive if the total IFN-γ-producing SFCs against peptides tested were ≥30 per 2 × 10^5^ input cells.

### Immunofluorescence staining and FACS analysis

For experiments shown in Figures 7 and 8, immunofluorescence staining was performed at 24 h and 3 or 4 days of stimulation as described previously^59^ using anti-human IgG fluorochrome-coupled antibodies against CD3 (clone OKT3, APC), CD4 (clone RPA-T4, FITC), CD8 (clone RPA-T8, PE), CD56 (clone 5.1H11, FITC), CD19 (clone 4G7, PE), and CD69 (clone FN50) (all from BioLegend). AAV.RBD VLP-stimulated PBMCs were stained with antibodies to human CD3, CD4, CD8, CD45RA, CD62L programmed death-1, cytotoxic T-lymphocyte-associated protein 4, and lymphocyte activation gene-3. Concentrations were used according to the manufacturer’s instructions. Normal mouse serum (3%) was added to block unspecific Fc-receptor binding. After staining, cells were fixed with 1% paraformaldehyde/PBS and stored at 4°C until data acquisition with FACScalibur (BD Biosciences). Data analysis was performed with FlowJo 10.5.0 software (BD Biosciences) or MRFLOW software. Fluorescence cut off was defined by respective isotype controls (BioLegend). T cell subsets were defined as follows: naive, CD3^+^CD45RA^+^CD62L^+^; effector memory, CD3^+^CD45RA^−^CD62L^−^; central memory, CD3^+^CD45RA^−^CD62L^+^; and terminally differentiated effector memory, CD3^+^CD45RA^+^CD62L^−^.

### Thermal stability assay

The thermal stability of the AAV vectors was measured using differential scanning fluorimetry with the Prometheus NT.48 (NanoTemper Technologies, Munich). AAV vectors (10 μL) were loaded into capillary tubes and heated at a linear temperature gradient (1°C/min) from 20°C to 100°C. The fluorescence signal at 330 and 350 nm was measured, corresponding to tryptophan residues that increasingly become exposed upon capsid unfolding. The melting temperature is indicated by the inflection point of the fluorescence ratio (330/350 nm) or the maximum of its first derivative.

### Statistics

If not mentioned otherwise, *n* = 3 biological and/or technical replicates were performed. Statistical significance was evaluated using GraphPad Prism 9.1 (GraphPad, San Diego). Either paired or unpaired two-way analysis of variance was performed following Sidak’s or Tukey’s post-hoc multiple comparisons test. The results are shown as mean ± standard error of the mean (SEM). Only values of *p* ≤ 0.05 were accepted and considered significant. Levels of significance: ∗*p* ≤ 0.05, ∗∗*p* < 0.01, ∗∗∗*p* < 0.001.

## Data and code availability

The authors confirm that the data supporting the findings of this study are available within the article and its supplemental information.

## Acknowledgments

We would like to thank Kerstin Skokann and Johanna Koch (both from LMU) for their technical assistance, and Jacqueline Bogedein (LMU) for help during the initial phase of the project. We also thank Prof. Elfriede Nößner and Barbara Mosetter (Helmholtz Center, Munich) for help with the bioplex analysis. Illustrations in Figures 4, 5, and S1 were created with BioRender.com. The molecular graphics were performed with UCSF Chimera, developed by the Resource for Biocomputing, Visualization, and Informatics at the University of California, San Francisco, with support from NIH P41-GM103311. This work was supported by grants from the Deutsche Forschungsgemeinschaft (DFG) to S.M. (SPP2127, project MI 1238/4-1).

## Author contributions

S.M. designed the AAV.RBDs and initiated the project. S.M., E.Y., and G.W. analyzed the data and supervised the experiments. S.B. performed molecular biology, cell culture, microscopy, dot blot experiments, analytics, and analyzed the data. M.G., M.D.-M., A.P., and M.G. performed and analyzed PBMC experiments, cytokine assays, and FACS. L.H. performed molecular biology. B.F. performed cell culture experiments. C.K. performed *in silico* modeling and contributed to analytics, molecular biology, and dot blot experiments. A.O. performed chemiluminescent microparticle immunoassays to detect SARS-CoV2-spike-specific antibodies in human plasma. H.A. designed and performed the rabbit studies, antibody measurements, and related ELISA. G.W. and E.Y. supervised, designed, and analyzed immunological experiments. S.M., S.B., and G.W. wrote the manuscript. All authors contributed to manuscript writing.

## Declaration of interests

S.M., S.B., L.H., and H.A. are listed inventors on the related patent application PCT/EP2021/074987 covering the novel AAV engineered capsids.

## Supplemental information

Document S1. Figures S1–S4, Tables S1, and S2

Document S2. Article plus supplemental information
