# Supplementary material for: A novel platform for engineered AAV-based vaccines
Source: Mol Ther Methods Clin Dev. 2025 Jan 22;33(1):101418. doi: 10.1016/j.omtm.2025.101418 (PMC11850754; doi:10.1016/j.omtm.2025.101418)
Supplement: Document S1. Figures S1–S4, Tables S1, and S2 [file mmc1.pdf]

OMTM, Volume *33*

Supplemental information

A novel platform for engineered AAV-based vaccines

Sabrina Babutzka, Miranda Gehrke, Anastasia Papadopoulou, Maria Diedrichs-Möhring, Maria Giannaki, Lena Hennis, Bastian Föhr, Cale Kooyman, Andreas Osterman, Evangelia Yannaki, Gerhild Wildner, Hermann Ammer, and Stylianos Michalakis

Supplemental information

Table S1. RBD sequences. S1.1_RBD was used for AAV2.RBDv1 and AAV9.RBDv1 and S1.2_RBD for AAV2.RBDv2.

**Name**	**Protein sequence of insertion**
S1.1_RBD	TNLCPFGEVFNATRFASVYAWNRKRISNCVADYSVLYNSASFSTFKCYGVSPTKLNDLCFTNVYADSFVIRGDEVRQIAPGQTGKIADYNYKLPDDFTGCVIAWNSNNLDSKVGGNYNYLYRLFRKSNLKPFERDISTEIYQAGSTPCNGVEGFNCYFPLQSYGFQPTNGVGYQPYRVVVLSFELLHAPATVCGPKKG
S1.2_RBD	KCTLKSFTVEKGIYQTSNFRVQPTESIVRFPNITNLCPFGEVFNATRFASVYAWNRKRISNCVADYSVLYNSASFSTFKCYGVSPTKLNDLCFTNVYADSFVIRGDEVRQIAPGQTGKIADYNYKLPDDFTGCVIAWNSNNLDSKVGGNYNYLYRLFRKSNLKPFERDISTEIYQAGSTPCNGVEGFNCYFPLQSYGFQPTNGVGY

Table S2. Demographic data of blood donors.

	**Age**	**Gender**	**Site**	**Data shown in**
Donor 1	62	female	Munich	Figures 5, 6 and 7
Donor 2	46	male	Munich	Figures 5, 6 and 7
Donor 3	62	male	Munich	Figures 5, 6 and 7
Donor 4	47	male	Thessaloniki	Figure 8
Donor 5	26	female	Thessaloniki	Figure 8
Donor 6	52	female	Thessaloniki	Figure 8

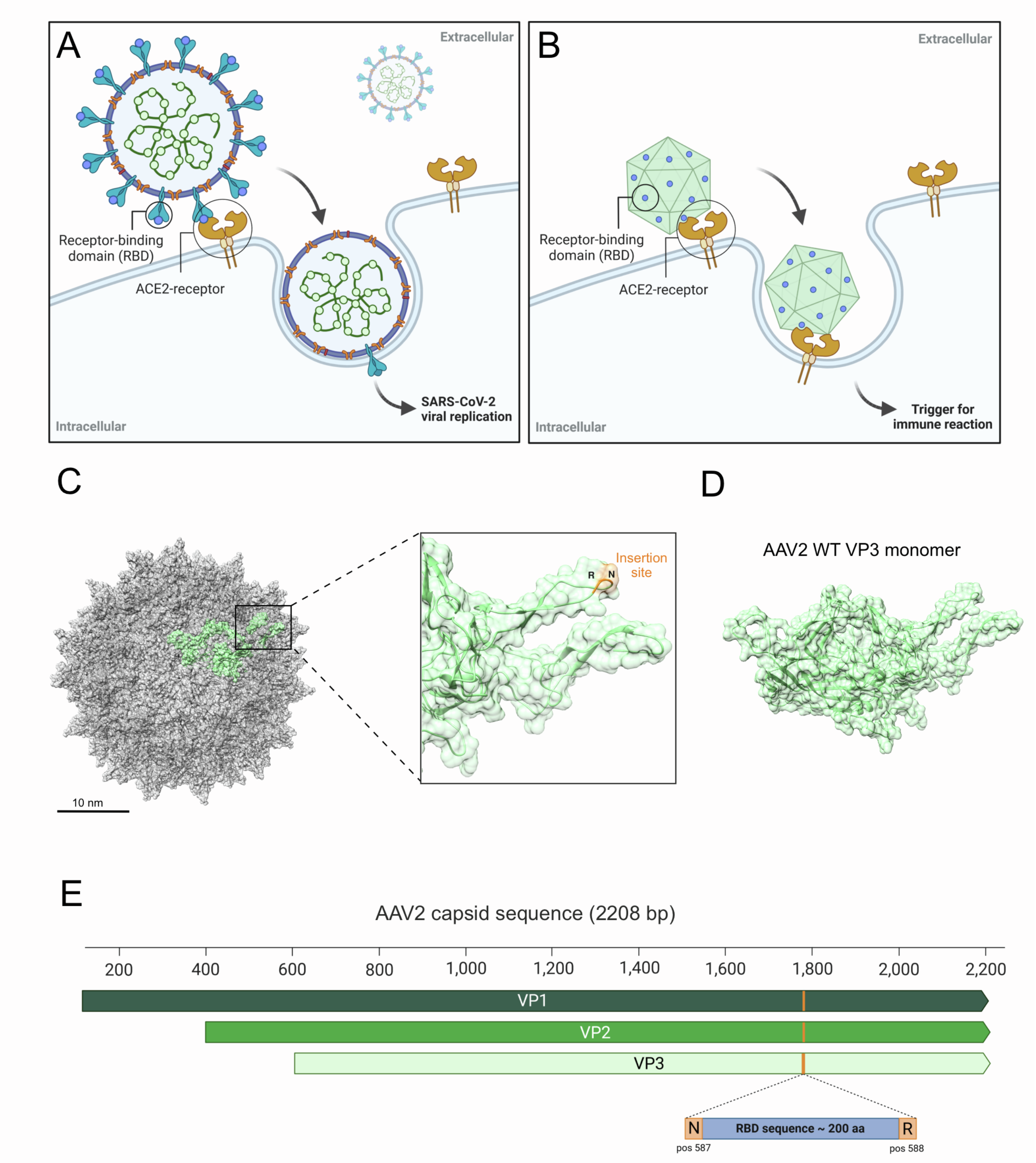

Figure S1. Novel AAV capsids with large peptide insertions. (A) Schematic interaction between SARS-CoV-2 viral particle and ACE2-receptor on human cells (B) Modified AAV capsid presenting the RBD of SARS-CoV-2 on the capsid surface. (C) Biological assembly of AAV2 WT capsid (PDB: 6ih9) showing the location of the insertion site in loop IV on the capsid surface. (D) Single VP3 monomer comprising the AAV capsid (green). (E) Location of the peptide insertion (blue) in the AAV2 cap sequence between N587 and R588. ACE2 angiotensin-converting enzyme, RBD receptor-binding domain.

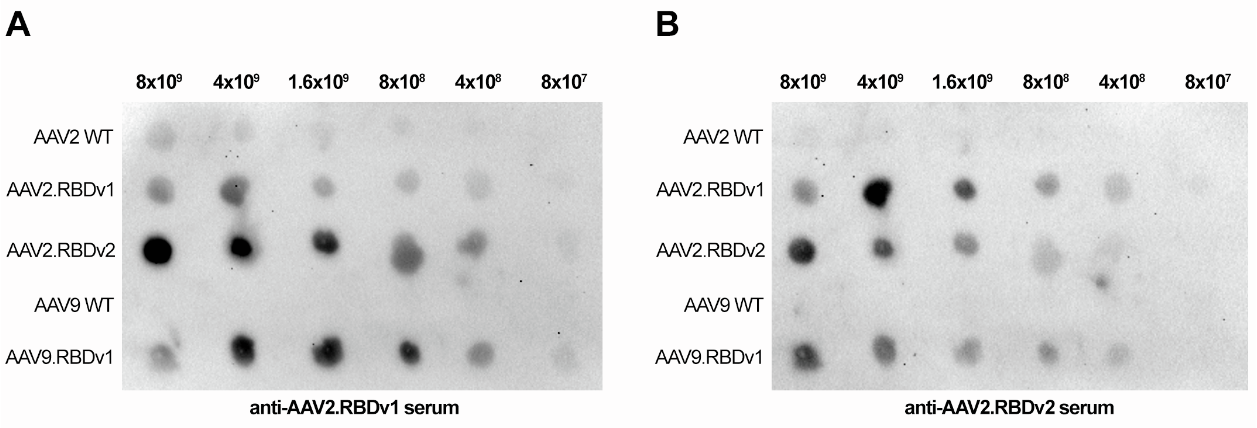

Figure S2. Dot blot analysis of anti-AAV2.RBDv1 and anti-AAV2.RBDv2 sera. (A-B) AAV.RBD and AAV WT capsid variants were spotted on PVDF membranes at indicated total capsid particle amount. The dot blots were probed with (A) serum of from an AAV2.RBDv1-immunized rabbit (at 1:2 000 dilution) and (B) serum of from an AAV2.RBDv2-immunized rabbit (at 1:2 000 dilution). The serum shows a strong reactivity with all AAV.RBD variants.

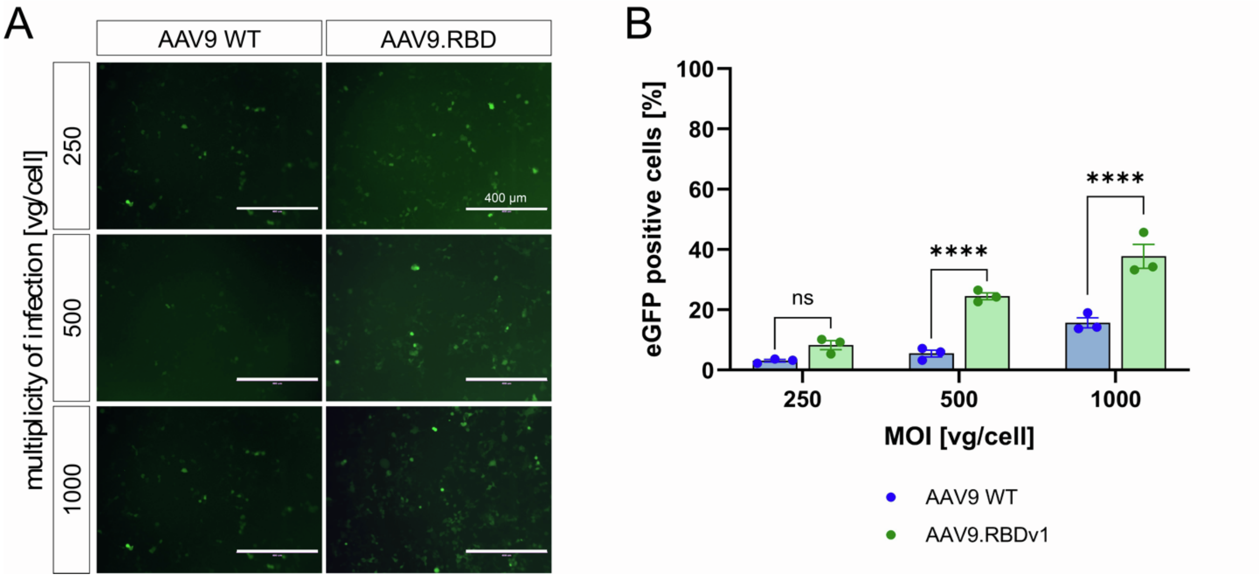

Figure S3. Transduction properties of AAV9 WT and AAV9.RBDv1 in ACE2-OE-HEK293T cells. (A) Representative epifluorescence images of HEK293T cells stably overexpressing ACE2 (ACE2-OE-HEK293T) captured at 48 h after infection at indicated multiplicities of infection (MOIs) with AAV9 WT or AAV9.RBDv1 expressing eGFP from an scCMV-eGFP genome. Scale bar marks 400 µm. (B) Quantification of the fraction of eGFP-positive cells. Images and quantification were acquired 48 h post infection. 2-way ANOVA, Šídák's multiple comparisons test: **** $p < 0.0001$, error bars indicate ± SEM, n = 3.

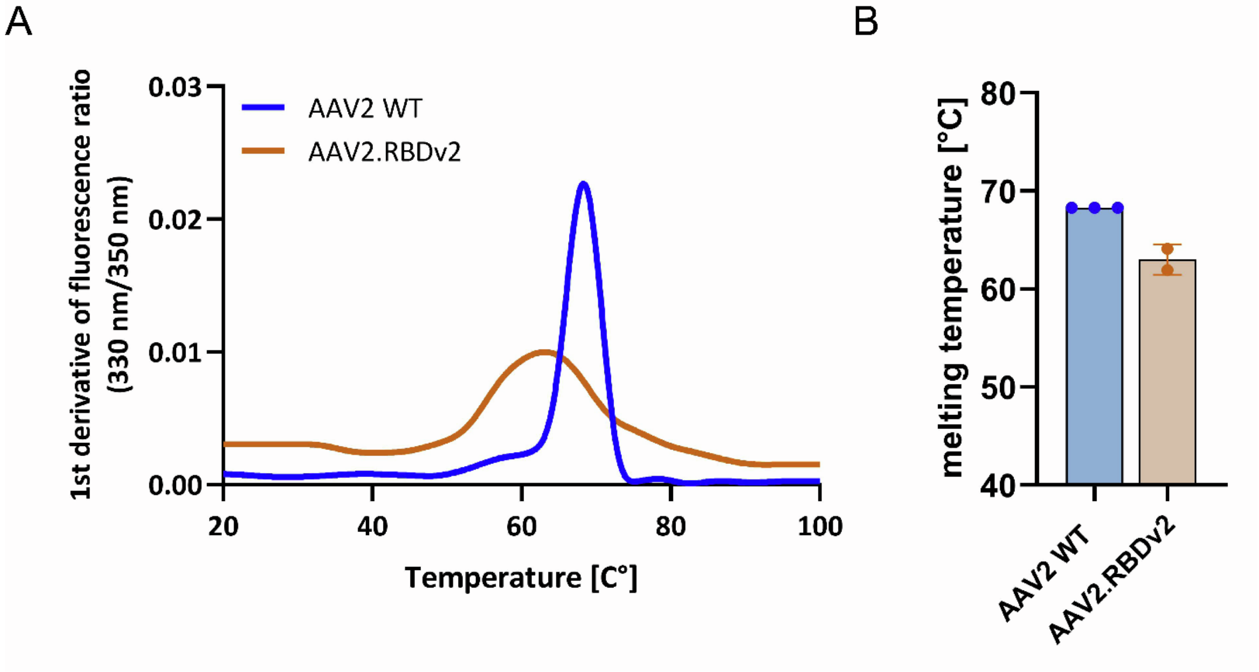

Figure S4. Thermal stability of AAV2.RBDv2 measured using differential scanning fluorimetry. (A) The first derivative of the ratio between the fluorescence at 330 and 350 nm at increasing temperatures. (B) Calculated melting temperatures of AAV2 vectors based on (A). n = 3 for AAV2 WT, n = 2 for AAV2.RBDv2.
